# Prolapsus of an Ovary

**Published:** 1863-03

**Authors:** L. R. Holmead

**Affiliations:** Chicago


### PROLAPSUS OF AN OVARY.

By L. R. HOLMEAD, M. D., Chicago.

On July 21, 1861, I was called to see Mrs. B——p, aged 30 years, who has given birth to six living children, and had seven miscarriages. Her general health has been bad for several months. I found her in great pain, which she described as a bearing down in the lower part of the abdomen, and more severe than any labor she had ever passed through.

About six weeks previous she had a miscarriage, with considerable loss of blood; and was just getting about. Her bowels being costive, she had the day before taken some cathartic medicine; and while at stool, felt something give way, which gave her such great pain that she could not help screaming. After taking a dose of opium she went to bed and was easy until the next morning, when the pain returned, with a constant desire for a movement of the bowels, but from the distress it gave her she was compelled to resist the inclination.

Fearing a misplacement of some of the organs of generation, I requested an examination, which she declined; I concluded to place her upon full doses of Opium, and Ol. Terebinth, gtts. viij, every 3 hours.

22nd—8 A. M. Pulse good. Tympanitis subsiding. Passed a comfortable night. Continued treatment. 10 A. M. Pain returned. Pulse very frequent and weak. Abdomen distended enormously. To the former treatment ordered warm fomentations, which gave some relief. Declined attending the case longer unless an examination was granted me, or, the privilege of calling counsel. Her husband suggested Dr. ———, and stating if we could not agree, that Dr. ——— should treat the case, and that I should continue my visits, and be released from all responsibilities. Dr. ———, upon making an examination, pronounced it an abscess in the cellular tissue between the Rectum and Vagina, just below the Os Uteri. I asked him how an abscess could cause such sudden trouble, he gave me no definite answer, but said that there could be no mistake about it, as he had had at least a dozen such cases that season. He described it as being a hard, immovable tumor, and thought that he could detect fluctuation.

Not being satisfied with his diagnosis, I yielded the treatment of the case to him, and continued my visits. He ordered the following: ℞. Quinine Sulph. ʒss.; Ext. Hyoscyami, gr. xv; Ft. Pil. No. 15; S. one every two hours. Warm fomentations to be continued.

23rd—Continues about the same. Treatment continued.

24th—No improvement. Dr. ——— ordered ℞. Hydrarg. Chlo. Mit. Pulv. Opii. a a gr. v; Pulv. Ipecac, gr. iv; M. Ft. Pil. No. 10; S. one every 3 hours.

The same course of treatment was continued until the 29th, Dr. ——— visiting the patient in the mean time. No improvement in condition of patient, except occasional relief from pain for a short time. Dr. ——— expecting every day that the abscess would be in a condition to puncture.

Mr. B——p becoming dissatisfied, I proposed to Dr. ——— to call counsel, which he thought not necessary, and stated that he was positive that he felt fluctuation, and that the abscess would break in a day or two.

He ordered: ℞. Hydrarg. Chlo. Mit., Pulv. Opii, a a gr. v; M. Ft. Pil. No. 10. S. one every 2 hours. ℞. Liq. Ammoniæ; Ol. Terebinth, a a ℥j; Ol. Olivæ ℥ij; M. S. use as a liniment over the abdomen.

30th—4 A. M.—Mr. B——p called upon me, saying something must be done for his wife, as her sufferings were greater than ever, and wished me to take the case in my own hands; which I declined, but proposed calling in Prof. J. Adams Allen, who was thereupon invited to see the case the same day. On digital examination, he found the hard tumor in the position above indicated. From its mobility, and the history of the case, suspecting its true character, after manipulating for a few minutes he had the pleasure of feeling it disappear from before the fingers, with immediate relief to the patient.

She was ordered a mild saline laxative and a pill of Hyoscyamus.

The laxative operated readily, but there was no further occasion for the pill. The patient convalesced immediately. Since, she has, upon two or three occasions, experienced some recurrence of the original difficulty, but a few hours confinement to the recumbent position has been followed by complete relief.

The right ovary, enlarged and heavy, had prolapsed into the recto-vaginal space, thus presenting to digital examination a well defined tumor.

The rarity of the accident, and the ease with which it was relieved, seem to render the case worthy of record.

The apparent fluctuation possibly depended upon a cyst in the ovary.

Similar cases are referred to by authors: (Vide Blundell Dis. of Women, p. 108. J. B. Brown, ditto, p. 179.)

The ultimate prognosis of the case is, of course, doubtful, as the displacement may recur at any time, or result in oppressive, or even fatal growth of the ovarian tumor.

